# [Corrigendum] Adipose-derived stem cell sheets combined with β-tricalcium phosphate/collagen-I fiber scaffold improve cell osteogenesis

**DOI:** 10.3892/etm.2025.12993

**Published:** 2025-10-13

**Authors:** Yang Wang, Xiaojia Song, Rui Lei, Ning Zhang, Liangping Zhang, Wei Xiao, Jinghong Xu, Jun Lin

Exp Ther Med 21:452, 2021; DOI: 10.3892/etm.2021.9882

Subsequently to the publication of the above article, the authors contacted the Editor to explain that they had selected an incorrect image to show the β-tricalcium phosphate/collagen-I fiber scaffolds with scattered adipose-derived stem cell sheets in Fig. 5B on p. 6; essentially, this image originated from a similar experiment that was being performed at the same time with another type of scattered stem cells.

However, the authors retained their original data, and a revised version of Fig. 5, now showing the correct data for Fig. 5B, is shown opposite. Note that the error made in assembling Fig. 5 did not have a major impact on either the overall results or on the conclusions reported in this study. All the authors agree with the publication of this corrigendum, and are grateful to the Editor of *Experimental and Therapeutic Medicine* for granting them the opportunity to publish this; furthermore, they apologize to the readership for any inconvenience caused.

## Figures and Tables

**Figure 5:**
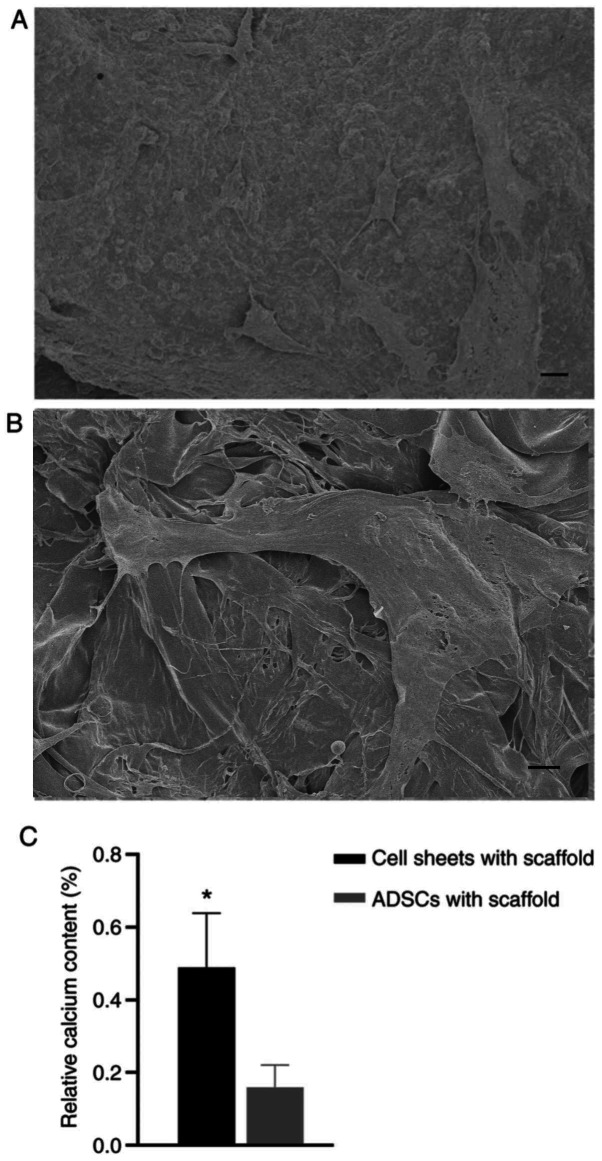
Surface morphologies of two types of complexes visualized by scanning electron microscopy after osteogenic induction for 13 days. (A) β-TCP/COL-I scaffolds with ADSCs sheet (scale bar, 10 µm). (B) β-TCP/COL-I scaffolds with scattered ADSCs (scale bar, 10 µm). (C) Relative calcium content on the surface of the two composites. ^*^P<0.05 vs. ADSCs with scaffold. ADSC, adipose-derived stem cell; β-TCP/COL-I, β-tricalcium phosphate/collagen fiber.

